# Clostridium Abundance and Lower Weight-for-Age z Scores Among 6-Month-Old Infants: Nested Cross-Sectional Study

**DOI:** 10.2196/87452

**Published:** 2026-07-06

**Authors:** Syamsul Alam, Veni Hadju, Ansariadi Ansariadi, Nurhaedar Jafar, Muh Tahir Abdullah, Syamsuar Manyullei

**Affiliations:** 1 Faculty of Public Health, Hasanuddin University, Makassar, South Sulawesi, Indonesia; 2 Department of Public Health, Faculty of Medicine and Health Sciences, Universitas Islam Negeri Alauddin, Makassar, South Sulawesi, Indonesia; 3 Department of Nutrition, Faculty of Public Health, Hasanuddin University, Tamalanrea, Makassar, South Sulawesi, 90245, Indonesia, 62 811441803; 4 Department of Epidemiology, Faculty of Public Health, Hasanuddin University, Makassar, South Sulawesi, Indonesia; 5 Center of Epidemiology, Faculty of Public Health, Hasanuddin University, Makassar, South Sulawesi, Indonesia; 6 Department of Biostatistics, Reproductive Health and Family Planning, Faculty of Public Health, Hasanuddin University, Makassar, South Sulawesi, Indonesia; 7 Department of Environmental Health, Faculty of Public Health, Hasanuddin University, Makassar, South Sulawesi, Indonesia

**Keywords:** *Clostridium*, gut microbiota, weight-for-age *z* score, infants, Indonesia

## Abstract

**Background:**

The gut microbiota plays a crucial role in infant nutrition through its effects on energy metabolism, nutrient absorption, and immune regulation. However, evidence from Indonesian infants remains limited.

**Objective:**

This study aimed to examine the association between genus-level gut microbiota abundance and weight-for-age *z* scores (WAZ) among 6-month-old infants in coastal Banggai District, Central Sulawesi, Indonesia.

**Methods:**

We conducted a nested follow-up cross-sectional observational analysis of 88 six-month-old infants, including 42 (47.7%) who were born to mothers who were assigned to receive *Moringa oleifera* enriched with royal jelly group and 46 (52.3%) who were assigned to receive a multiple micronutrient supplement in a previous maternal supplementation trial. Maternal and infant characteristics were collected via structured interviews and standardized anthropometric measurements. WAZ was calculated using the World Health Organization Child Growth Standards, and underweight (WAZ <−2 SD) was reported as a secondary indicator. Stool samples were analyzed using genus-specific quantitative polymerase chain reaction to quantify *Bifidobacterium*, *Lactobacillus*, *Bacteroides*, *Clostridium*, and *Escherichia coli* (log_10_ colony-forming unit/mL). Associations between bacterial abundance and WAZ were assessed using multivariable linear regression adjusted for maternal supplementation allocation and relevant maternal, environmental, and infant covariates.

**Results:**

The pooled mean WAZ was −0.47 (SD 1.09), and 8% (7/88) of the infants were underweight. The combined abundance of beneficial genera was higher than that of opportunistic bacteria (*E coli* and *Clostridium;* Wilcoxon signed-rank test; *P*=.002). Higher *Clostridium* abundance was inversely associated with WAZ (unadjusted β=−.094, 95% CI −0.173 to −0.015; *P*=.02; adjusted β=−.091, 95% CI −0.172 to −0.010; *P*=.03). No statistically significant associations were observed for *Bifidobacterium* (*P*=.13), *Lactobacillus* (*P*=.19), *Bacteroides* (*P*=.70), or *E coli* (*P*=.18) in adjusted models.

**Conclusions:**

Among 6-month-old infants in coastal Central Sulawesi, higher genus-level *Clostridium* abundance was independently associated with lower WAZ. Given the cross-sectional design and genus-level quantitative polymerase chain reaction assessment, temporality and species-level mechanisms cannot be established. Longitudinal studies using more comprehensive microbiome profiling are warranted to clarify potential pathways linking gut microbiota and early-life growth.

## Introduction

The growth of infants aged 0 to 6 months represents a critical period that is strongly influenced by nutritional status. One of the most sensitive indicators for assessing nutritional status is the weight-for-age *z* score (WAZ), where values below −2 SD are classified as underweight. Undernutrition, which may result from stunting, wasting, or both, is reflected by being underweight and contributes substantially to morbidity, mortality, and long-term developmental impairments in early life [1,2].

Globally, the prevalence of underweight among children under 5 has declined considerably over the past decades. According to the World Bank’s World Development Indicators, derived from the Joint Child Malnutrition Estimates, the global prevalence has decreased from approximately 25% in the early 1990s to around 12% to 13% in recent years [3]. In Indonesia, the 2024 Indonesian Nutrition Status Survey reported a prevalence of 14.5%, showing a decline from the previous year but still above the national target [4]. At the regional level, disparities persist, with the 2023 Indonesian Health Survey reporting an underweight prevalence of 24.4% in Central Sulawesi and 22.7% in Banggai District [5]. This prevalence exceeds the World Health Organization (WHO) threshold for a high public health burden (20%‐29%), indicating a persistent challenge for child nutrition in these areas.

Although these figures illustrate the magnitude of the problem, understanding the underlying biological and environmental determinants remains essential. In addition to inadequate dietary intake and infectious diseases, recent scientific evidence has highlighted the critical role of the gut microbiota in shaping infant nutritional status. The gut microbiota influences energy metabolism, nutrient absorption, and immune regulation [6,7]. Emerging studies have shown that early-life variations in microbiota composition can affect linear growth and the risk of undernutrition [8]. Reviews have also emphasized the close link between microbial diversity, immune system maturation, and overall infant health [9,10].

Several studies have demonstrated associations between gut microbiota dysbiosis and growth impairment. Research in Bangladesh and Malawi found that children with growth faltering generally had lower levels of *Bifidobacterium* and *Lactobacillus*, along with a higher abundance of opportunistic bacteria such as *Enterobacteriaceae* [11-13]. Similarly, a study in Finland reported that probiotic supplementation could restore microbiota balance and promote infant growth [14]. However, evidence from Indonesia remains limited, particularly in coastal regions such as Central Sulawesi, where nutritional challenges and environmental exposures may influence early-life microbial colonization.

Moreover, most previous studies have primarily focused on stunting, while limited research has examined the association between gut microbiota balance and WAZ during early infancy. Therefore, this study aimed to examine the association between genus-level gut microbiota abundance and WAZ among 6-month-old infants in coastal Banggai District, Central Sulawesi, Indonesia.

## Methods

### Study Design and Setting

This study was a nested follow-up cross-sectional observational analysis of infants born to mothers who had previously participated in a randomized maternal supplementation trial conducted in coastal areas of Banggai District, Central Sulawesi, Indonesia, specifically in the rural subdistricts of Batui Selatan and Moilong. In the original trial, pregnant women were randomly assigned to receive either multiple micronutrient supplements (MMS) in accordance with national guidelines or *Moringa oleifera* enriched with royal jelly (MRJ) during pregnancy. Supplementation was provided from the second trimester until delivery (September 2023-June 2024).

This study used follow-up data from the infant cohort to examine the association between gut microbiota composition and infant nutritional status at 6 months of age, measured using WAZ. Because the infants originated from the previous maternal supplementation trial, the original supplementation allocation (MRJ vs MMS) was considered in the statistical analysis to account for potential confounding. This study did not evaluate the efficacy of the maternal supplementation intervention and did not involve new randomization or intervention during infancy. The original trial was not registered in a public trial registry; therefore, this manuscript is reported as a nested observational follow-up analysis rather than as a primary randomized trial report.

Follow-up data collection was conducted between August and December 2024 through household visits. Anthropometric measurements and questionnaire-based information on maternal, infant, and environmental characteristics were obtained, and infant fecal samples were collected for microbiota analysis. Laboratory analyses of the fecal samples were completed by April 2025 at a university-affiliated medical research center. Reporting followed the STROBE (Strengthening the Reporting of Observational Studies in Epidemiology) guideline for cross-sectional studies because this manuscript reports a nested observational follow-up analysis rather than the primary outcomes of the original randomized trial (Checklist 1).

### Participants

Infants included in this study were born to mothers who had participated in the original randomized controlled trial of maternal supplementation. In that trial, 160 pregnant women were randomly assigned to receive either the MRJ supplement—a daily capsule containing *M oleifera* leaf extract enriched with royal jelly—or a standard MMS in accordance with national guidelines. Supplementation was provided from the second trimester until delivery (September 2023-June 2024).

During the follow-up phase (August-December 2024), infants were assessed when they reached 6 months of age. The follow-up analysis focused specifically on infants at 6 months of age. Infants who had already exceeded 6 months of age at the time of follow-up or whose families had relocated outside the study area were not included in the analysis. Because the outcome assessment was restricted to infants aged 6 months, anthropometric measurements were not obtained for those who were not eligible for follow-up. A total of 88 infants met the inclusion criteria and were enrolled in this analysis (n=42, 47.7% from the MRJ group and n=46, 52.3% from the MMS group).

### Ethical Considerations

This study was approved by the ethics committee of the Faculty of Public Health, Hasanuddin University (protocol code 281024093076; approval 3505/UN4.14.1/TP.02.02/2024). Written informed consent was obtained from all participants prior to enrollment.

### Sample Size Considerations

The sample size for this nested follow-up analysis was determined by the number of infants who met the predefined eligibility criteria (assessment at 6 months of age) and were available during the follow-up period, rather than by an a priori sample size calculation for microbiota outcomes. To contextualize statistical power, a post hoc assessment indicates that with 88 participants and a 2-sided α level of .05, the study has approximately 80% power to detect a moderate association (equivalent to a correlation of *r*=0.30) in unadjusted analyses. We acknowledge that power may be lower in multivariable models after adjustment for multiple covariates; therefore, smaller associations may not have been detected, and nonsignificant findings for some genera should be interpreted cautiously.

### Variables and Outcomes

The primary outcome was infant nutritional status assessed using WAZ based on the WHO Child Growth Standards (2006) and analyzed as a continuous variable. Underweight (WAZ<−2 SD) was defined and reported as a secondary descriptive indicator. The primary exposure variable was gut microbiota composition at 6 months of age, focusing on 5 bacterial genera: *Bifidobacterium*, *Lactobacillus*, *Bacteroides*, *Clostridium*, and *Escherichia coli*. Potential covariates included infant sex, birth weight, mode of delivery, maternal age and education, household socioeconomic status, breastfeeding practices, and the maternal supplementation group (MRJ or MMS).

### Data Collection

Data were collected during home visits conducted by the research team in collaboration with trained enumerators. Structured questionnaires were administered to obtain information on maternal and infant characteristics, feeding practices, and sociodemographic factors. Following the interviews, anthropometric measurements of the infants were taken using calibrated equipment and standardized procedures, with assistance from the enumerators. In addition, fecal samples were collected from the infants for subsequent microbiota analysis.

### Anthropometric Measurements

Anthropometric assessments were performed when infants reached 6 months of age. Weight was measured using a calibrated digital infant scale, and recumbent length was recorded as supplementary information. Measurements were taken twice; if discrepancies were detected, a third measurement was performed, and the mean of the 2 closest values was used in the analysis. WAZ values were calculated using WHO Anthro software (version 3.2.2), and underweight was defined as WAZ<−2 SD according to WHO standards.

### Gut Microbiota Analysis

Fecal samples were stored at −20 °C and analyzed at the Hasanuddin University Medical Research Center. DNA was extracted using the Presto Stool DNA Extraction Kit (Geneaid Biotech Ltd) following the manufacturer’s protocol. Quantitative analysis of gut microbiota was performed using quantitative polymerase chain reaction (qPCR) with genus-specific primers targeting *Bifidobacterium, Lactobacillus, Bacteroides, Clostridium,* and *E coli*. Results were expressed as log_10_ colony-forming units (CFU)/mL. Additional laboratory details for DNA extraction and genus-level qPCR procedures are provided in Multimedia Appendix 1.

### Statistical Analysis

Data were analyzed using SPSS software (version 23; IBM Corp). Descriptive statistics were used to summarize maternal, infant, environmental, and microbiota characteristics. The Shapiro-Wilk test was applied to assess normality. Group comparisons between supplementation groups were performed using the chi-square test for categorical variables and the Mann-Whitney *U* test for continuous variables with nonnormal distributions. Paired differences in bacterial abundance within the same infants were assessed using the Wilcoxon signed-rank test. Associations between gut bacterial abundance (log_10_ CFU/mL) and WAZ were examined using multivariable linear regression models.

There were no missing data for variables included in the regression models; therefore, all regression analyses used n=88. Covariates were selected a priori based on biological plausibility and evidence from previous studies as potential confounders of the microbiota-growth relationship, including maternal supplementation group (MRJ vs MMS), maternal education, environmental health status, gestational age at birth, mode of delivery, infant illness history within the first 6 months, immunization status, exclusive breastfeeding, and birth weight category. To maintain parsimony given the modest sample size, we used this prespecified covariate set and fitted separate adjusted models for each bacterial genus (ie, without data-driven covariate selection procedures). Linear regression assumptions were evaluated using residual diagnostics for linearity and homoscedasticity and Q-Q plots for normality of residuals. Multicollinearity among covariates was assessed using variance inflation factors, and influential observations were examined using standardized residuals and the Cook distance. Model fit was summarized using *R*^2^ and adjusted *R*^2^. All tests were 2-tailed, and statistical significance was set at *P*<.05.

## Results

### Characteristics of Study Participants

**Figure 1.**
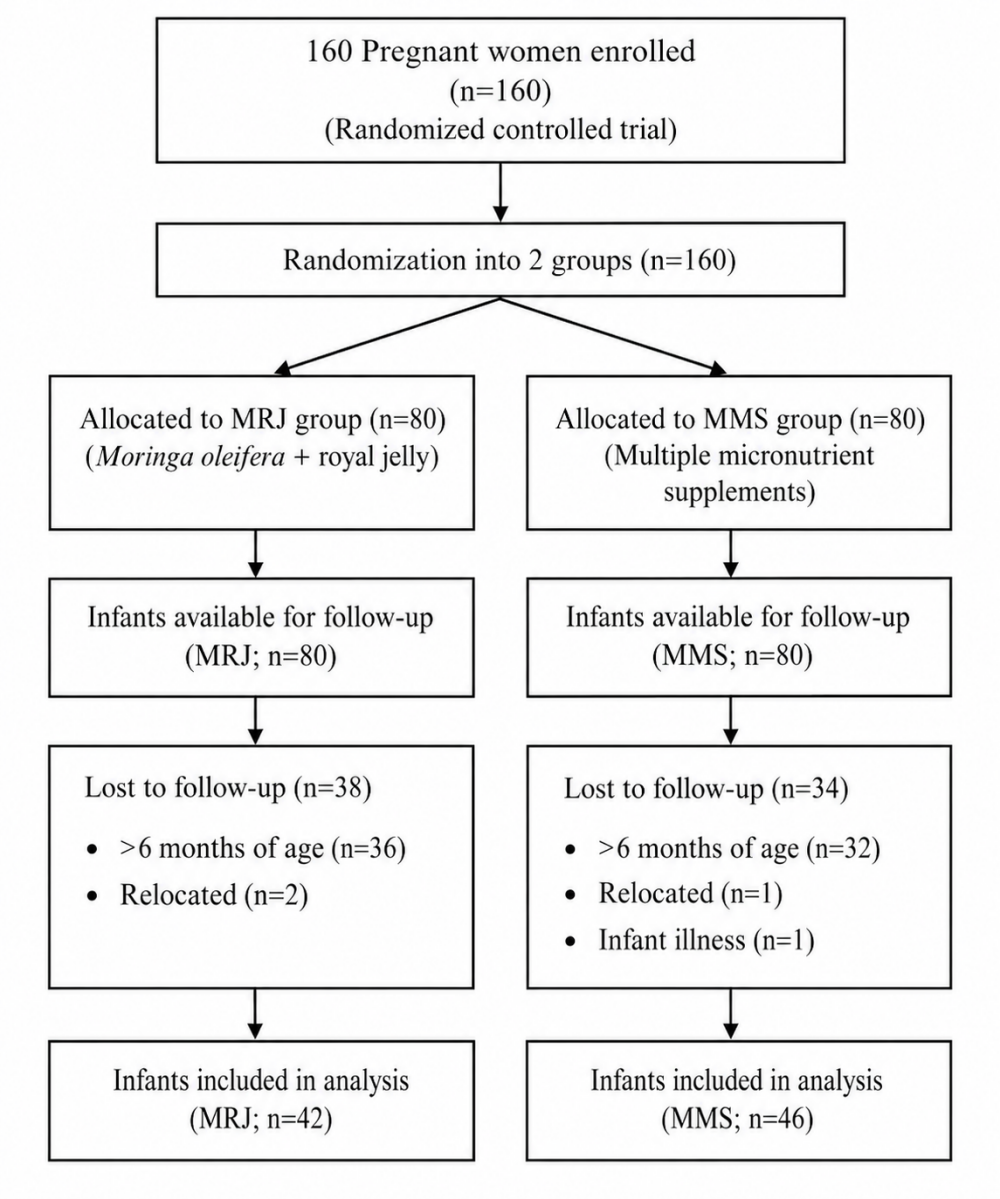
Flow diagram of study participants. MMS: multiple micronutrient supplements; MRJ: *Moringa oleifera* enriched with royal jelly.

A detailed participant flow diagram is presented in Figure 1. A total of 88 six-month-old infants were included in the analysis (n=42, 47.7% MRJ and n=46, 52.3% MMS). Baseline household, maternal, and infant characteristics are summarized in Table 1. Maternal and paternal education were categorized as low (primary and junior secondary school) and high (senior secondary school or higher, including diploma and university). Most baseline characteristics were comparable between groups; however, the MRJ group had a higher proportion of fathers with low education than the MMS group (15/42, 35.7% vs 6/46, 13%; *P*=.01), and households in the MMS group more often reported a drinking water source within ≤10 m (12/46, 26.1% vs 3/42, 7.1%; *P*=.02). Infant sex distribution also differed between groups (male infants: 26/46, 56.5% in MMS vs 14/42, 33.3% in MRJ; *P*=.03). Anthropometric outcomes at 6 months were similar between groups, including WAZ (*P*=.63). Overall, mean WAZ in the pooled sample was −0.47 (SD 1.09), and 8% (7/88) were classified as underweight. These pooled data were used in subsequent analyses to examine associations between gut microbiota composition and growth outcomes.

**Table 1.** Characteristics of study participants.

Variables	MRJ^a^ (n=42)	MMS^b^ (n=46)	Total (n=88)	*P* value
Sociodemographic characteristics, n (%)				
Maternal education				.05^c^
Low	10 (23.8)	4 (8.7)	14 (15.9)	
High	32 (76.2)	42 (91.3)	74 (84.1)	
Paternal education				*.01* ^c^
Low	15 (35.7)	6 (13)	21 (23.9)	
High	27 (64.3)	40 (87)	67 (76.1)	
Maternal occupation (unemployed)	32 (76.1)	35 (76.1)	67 (76.1)	.58^c^
Paternal occupation				.69^c^
Farmer	16 (38.1)	19 (41.3)	35 (39.8)	
Self-employed	7 (16.7)	4 (8.7)	11 (12.5)	
Household income <Banggai minimum wage	27 (64.3)	25 (54.3)	52 (59.1)	.34^c^
Environmental factors, n (%)				
Drinking water source ≤10 m	3 (7.1)	12 (26.1)	15 (17.0)	.*02*^c^
Adequate environmental sanitation	9 (21.4)	14 (30.4)	23 (26.1)	.24^c^
Maternal and perinatal characteristics, n (%)				
Infant sex				.*03*^c^
Male	14 (33.3)	26 (56.5)	40 (45.5)	
Female	28 (66.7)	20 (43.5)	48 (54.5)	
Preterm gestational age (<37 wk)	1 (2.4)	4 (8.7)	5 (5.7)	.20^c^
Delivery mode (C-section)	5 (11.9)	5 (10.9)	10 (11.4)	.57^c^
Maternal antibiotic use during pregnancy	12 (28.6)	15 (32.6)	27 (30.7)	.68^c^
Infant feeding practices, n (%)				
Early initiation of breastfeeding	17 (40.5)	23 (50)	40 (45.5)	.49^c^
Colostrum feeding	38 (90.5)	41 (89.1)	79 (89.8)	>.99^c^
Exclusive breastfeeding (0‐6 months)	22 (52.4)	15 (32.6)	37 (42)	.09^c^
Still breastfeeding at 6 months	34 (81)	38 (82.6)	72 (81.8)	>.99^c^
Anthropometric characteristics				
Low birth weight (<2500 g), n (%)	2 (4.8)	5 (10.9)	7 (8)	.35^c^
Birth weight (kg), mean (SD)	3.21 (0.43)	3.13 (0.54)	3.17 (0.49)	.48^d^
Birth length (cm), mean (SD)	48.9 (1.26)	48.6 (1.90)	48.7 (1.60)	.52^d^
Infant weight at 6 months (kg), mean (SD)	7.14 (0.93)	7.28 (0.86)	7.22 (0.89)	.45^d^
Infant length at 6 months (cm), mean (SD)	65.5 (2.5)	65.4 (3.3)	65.4 (2.9)	.79^d^
Weight-for-age *z* score, mean (SD)	−0.57 (1.12)	−0.36 (1.05)	−0.47 (1.09)	.63^d^
Underweight (weight-for-age *z* score <−2 SD), n (%)	4 (9.5)	3 (6.5)	7 (8)	.69^c^

a MRJ: *Moringa oleifera* enriched with royal jelly.

b MMS: multiple micronutrient supplements.

c Chi-square test.

d Mann-Whitney *U* test.

### Gut Microbiota Composition and Distribution by Nutritional Status in 6-Month-Old Infants

The gut microbiota composition of 6-month-old infants is summarized in Table 2. Among the beneficial bacterial genera, *Lactobacillus* exhibited the highest mean concentration (mean 7.83, SD 1.75 log CFU/mL), followed by *Bifidobacterium* (mean 7.44, SD 1.88 log CFU/mL). The commensal genus *Bacteroides* was detected at comparable levels (mean 7.52, SD 1.82 log CFU/mL). In contrast, the mean concentrations of opportunistic bacteria were 8.03 (SD 1.43) log CFU/mL for *E coli* and 6.00 (SD 2.73) log CFU/mL for *Clostridium*. When grouped, the combined abundance of beneficial bacteria (*Bifidobacterium* and *Lactobacillus*) was significantly higher than that of opportunistic bacteria (*E coli* and *Clostridium;* mean 7.64, SD 1.36 vs mean 7.01, SD 1.82 log CFU/mL; Wilcoxon signed-rank test: *Z*=1204.5; *P*=.002).

**Table 2.** Gut microbiota composition of 6-month-old infants (n=88, log colony-forming unit [CFU]/mL).

Gut microbiota	Values, mean (SD)	Values, median (IQR)
*Bifidobacterium* (beneficial)	7.44 (1.88)	7.69 (6.98‐8.51)
*Lactobacillus* (beneficial)	7.83 (1.75)	7.78 (6.50‐9.27)
*Bacteroides* (commensal)	7.52 (1.82)	7.54 (6.77‐8.26)
*Clostridium* (opportunistic)	6.00 (2.73)	7.03 (5.55‐8.12)
*Escherichia coli* (opportunistic)	8.03 (1.43)	7.84 (7.27‐8.70)
Beneficial bacteria^a^	7.64 (1.36)	7.69 (6.98‐8.51)
Opportunistic bacteria^a^	7.01 (1.82)	7.44 (5.89‐8.12)

a Wilcoxon signed-rank test (beneficial vs opportunistic bacteria): *Z*=1204.5; *P*=.002.

**Figure 2.**
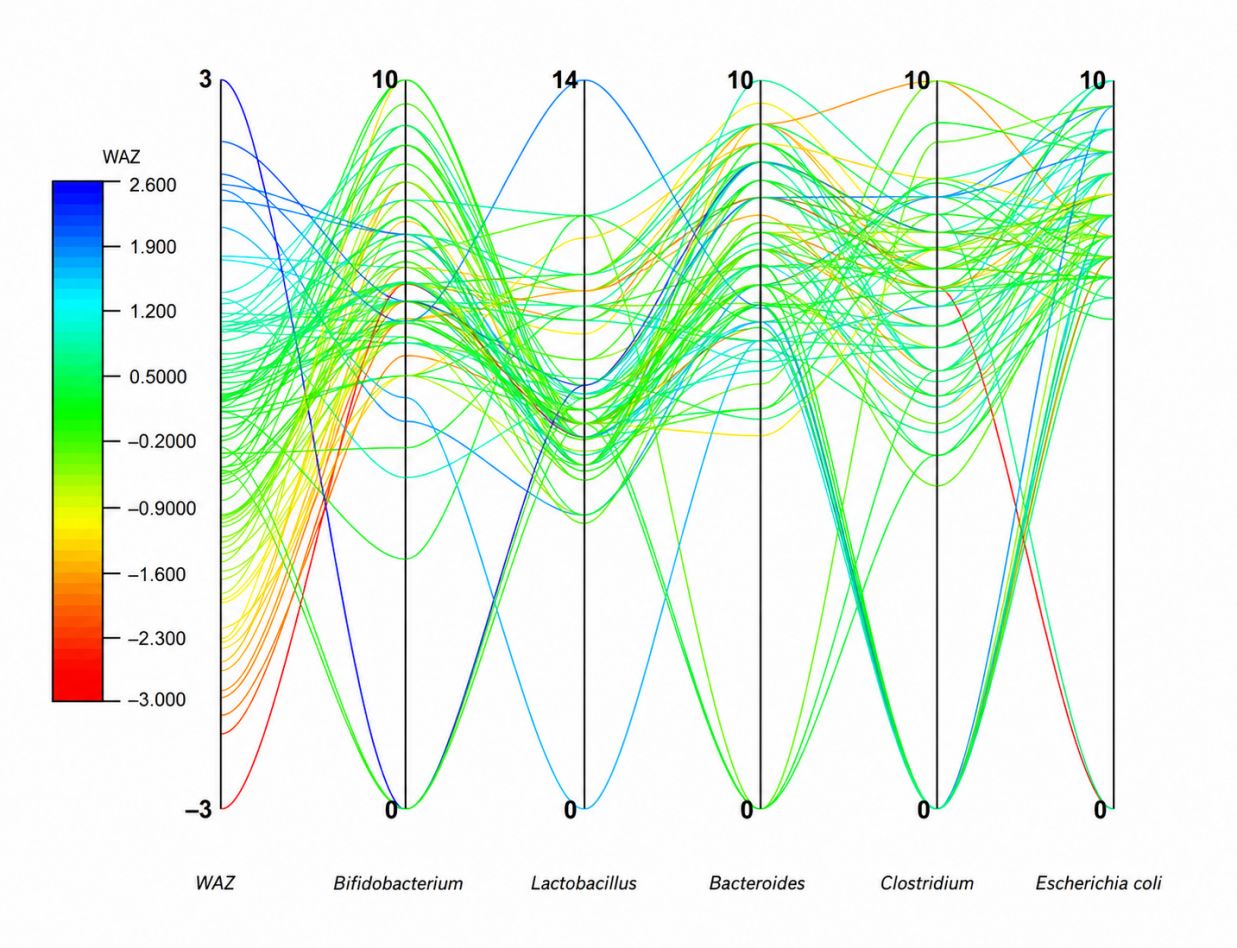
Parallel coordinates plot of genus-level gut microbiota abundance (log_10_ colony-forming unit [CFU]/mL) and weight-for-age *z* scores (WAZ) in 6-month-old infants (n=88). Each line represents one infant. Line color indicates WAZ (red=lower WAZ; blue=higher WAZ; observed range −3.0 to 2.6). Vertical axes show WAZ and bacterial genus abundance measured by quantitative polymerase chain reaction.

Figure 2 illustrates the distribution of the 5 dominant bacterial genera (*Bifidobacterium*, *Lactobacillus*, *Bacteroides*, *Clostridium*, and *E coli*) according to infant nutritional status. Each line in the figure represents an individual infant, and the color gradient indicates WAZ, ranging from very low values (red, <–3 SD) to high values (blue, approaching 2 SD). Infants with lower WAZ demonstrated greater variability in microbial abundance, particularly for *Lactobacillus* and *Clostridium*. Conversely, infants with normal or higher WAZ values exhibited more uniform distribution patterns across the analyzed genera. These results indicate that underweight infants tended to present more heterogeneous gut microbiota profiles, whereas well-nourished infants showed a more balanced bacterial composition.

### Association Between Gut Microbiota Genus and WAZ

**Figure 3.**
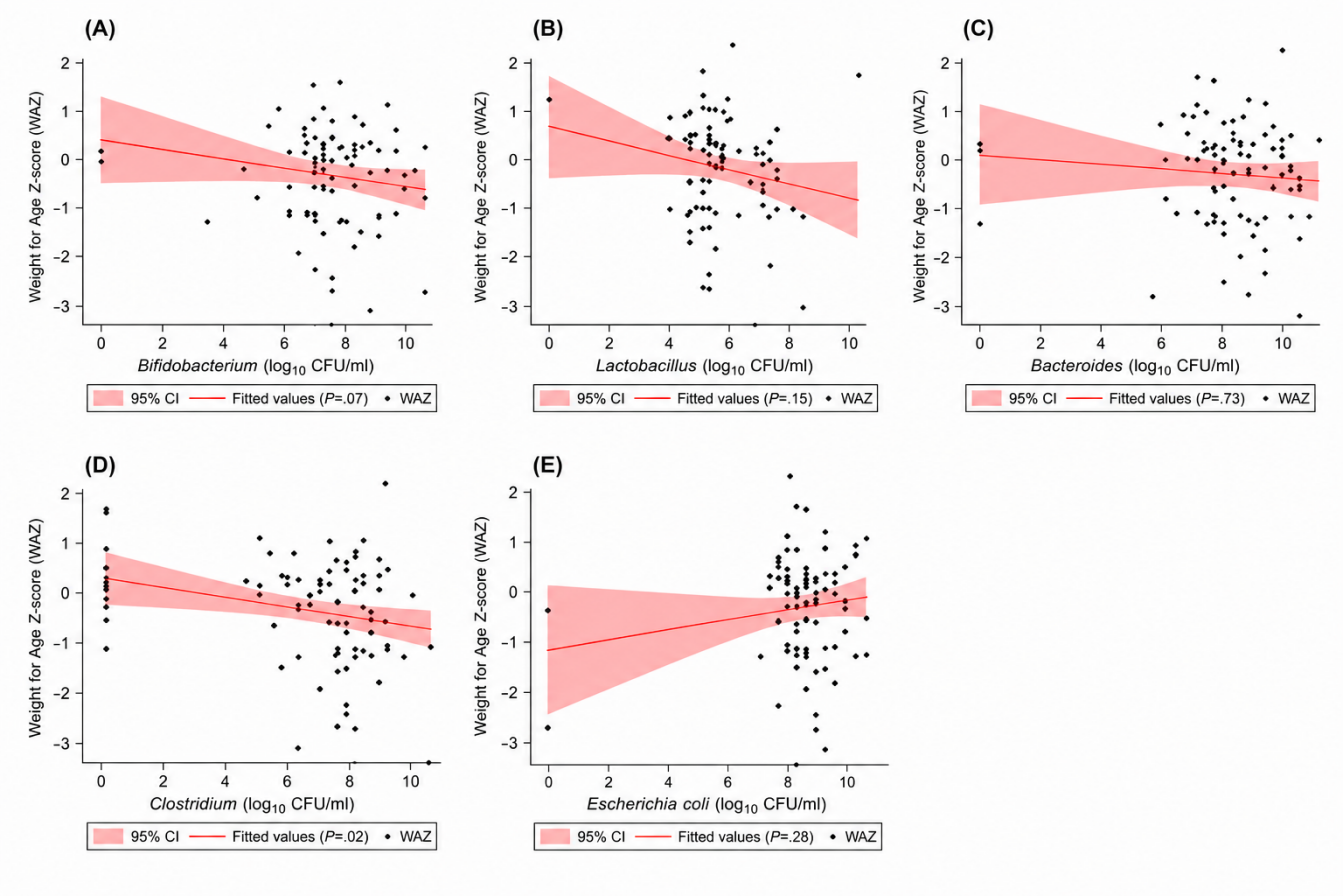
Scatterplots showing unadjusted associations between genus-level bacterial abundance (log_10_ colony-forming unit [CFU]/mL) and weight-for-age *z* scores (WAZ) in 6-month-old infants (n=88). Each point represents one infant. The solid line shows the fitted linear relationship and the shaded area represents the 95% CI for the fitted line. Panels (A-E) correspond to *Bifidobacterium, Lactobacillus, Bacteroides, Clostridium,* and *Escherichia coli*, respectively.

The associations between gut microbiota composition and WAZ are illustrated in Figure 3. Overall, the regression patterns indicated a predominantly negative trend between bacterial abundance and WAZ across most genera. For *Bifidobacterium* (panel A) and *Lactobacillus* (panel B), the regression lines exhibited a slight downward slope, suggesting that higher levels of these bacteria were associated with lower WAZ; however, these associations were not statistically significant. *Bacteroides* (panel C) showed a nearly flat regression line, indicating a weak relationship with WAZ.

In contrast, *Clostridium* (panel D) demonstrated a clear negative association, with a decreasing regression line, suggesting that greater *Clostridium* abundance was related to lower WAZ. For *E coli* (panel E), the regression line indicated a positive but nonsignificant trend, which should be interpreted with caution.

Table 3 summarizes the linear regression results examining the associations between bacterial genera and WAZ among 6-month-old infants. In the unadjusted model, none of the beneficial genera (*Bifidobacterium* and *Lactobacillus*) were significantly associated with WAZ, although both demonstrated a negative trend. Similarly, *Bacteroides* and *E coli* showed no significant associations. Conversely, *Clostridium* exhibited a significant negative association with WAZ (β=–.094, 95% CI –0.173 to –0.015; *P*=.02), indicating that higher *Clostridium* abundance was linked to lower weight-for-age status. This association remained significant in the adjusted model (β=–.091, 95% CI –0.172 to –0.010; *P*=.03) after controlling for maternal, perinatal, and environmental covariates. These findings indicate that higher *Clostridium* abundance was independently associated with lower WAZ, suggesting a potential link with early-life growth faltering.

**Table 3.** Association between gut microbiota genera and weight-for-age *z* scores (WAZ) in 6-month-old infants.

Genus	Model 1^a^		Model 2^b^	
	Unadjusted β (95% CI)	*P* value	Adjusted β (95% CI)	*P* value
*Bifidobacterium*	–0.105 (–0.220 to 0.010)	.07	–0.090 (–0.207 to 0.027)	.13
*Lactobacillus*	–0.091 (–0.215 to 0.033)	.15	–0.087 (–0.218 to 0.043)	.19
*Bacteroides*	–0.021 (–0.139 to 0.097)	.73	–0.024 (–0.149 to 0.101)	.70
*Clostridium*	–0.094 (–0.173 to –0.015)	.02^c^	–0.091 (–0.172 to –0.010)	.03^c^
*Escherichia coli*	0.083 (–0.067 to 0.233)	.28	0.110 (–0.051 to 0.271)	.18

a Model 1 (unadjusted): without confounding variables.

b Model 2 (adjusted): after controlling for confounding variables (maternal supplementation, maternal education, environmental health, gestational age, delivery process, infant illness history, immunization status, exclusive breastfeeding, and birth outcomes).

c *P*<.05.

## Discussion

### Principal Findings

This study found a significant inverse association between *Clostridium* abundance and WAZ among 6-month-old infants living in coastal areas of Central Sulawesi, Indonesia. This finding indicates that higher abundance of opportunistic bacteria was associated with lower WAZ and may be a marker of early-life growth faltering. In contrast, *Bifidobacterium*, *Lactobacillus*, *Bacteroides*, and *E coli* were not significantly associated with WAZ in the adjusted models. Although infants from both maternal supplementation groups (MRJ and MMS) exhibited a relatively low prevalence of underweight (7/88, 8%), mild growth faltering persisted, indicating that postnatal influences such as feeding practices, environmental exposures, and gut microbiota composition likely continue to shape infant growth outcomes beyond prenatal nutritional interventions. Because the microbiota assessment in this study included only 5 bacterial genera quantified by genus-level qPCR, interpretations should be limited to genus-level associations. Recent systematic evidence supports a broader link between gut microbiota composition and child growth outcomes, including growth faltering and stunting, although findings vary across settings and methods [15], and recent reviews emphasize the intersection of undernutrition, microbiome development, and early child health outcomes [16].

The gut microbiota plays a critical role in nutrient metabolism, immune regulation, and intestinal homeostasis during infancy. In this study, beneficial genera such as *Bifidobacterium* and *Lactobacillus* were predominant, consistent with their established roles in carbohydrate fermentation, production of short-chain fatty acids, and support of mucosal immunity [15-17]. These genera are often enriched in breastfed infants and are linked to favorable nutritional outcomes. However, neither *Bifidobacterium* nor *Lactobacillus* demonstrated significant associations with WAZ in this cohort, which may reflect limited variability within a generally adequate range and/or limited power to detect smaller effect sizes. The nonsignificant association observed for *Bifidobacterium* should be interpreted with caution and does not necessarily indicate that this genus is not relevant to infant growth. *Bifidobacterium* is widely recognized as a key early-life gut colonizer involved in human milk oligosaccharide metabolism, short-chain fatty acid production, gut barrier maturation, and immune development [16,18]. Recent evidence also supports the role of *Bifidobacterium* in infant immune development and highlights species- and strain-specific functional differences, which may not be captured by genus-level qPCR assessment [18,19]. In this study, the lack of a significant association may therefore be partly explained by the modest sample size, limited between-infant variability, cross-sectional assessment at a single time point, and the inability to distinguish species- or strain-level variation.

In contrast, *Clostridium* exhibited a robust negative association with WAZ after adjusting for maternal, perinatal, and environmental factors, including maternal supplementation allocation. This aligns with previous studies reporting that differences in the abundance of opportunistic taxa are associated with impaired intestinal barrier function, low-grade inflammation, and decreased nutrient absorption efficiency [18-21]. Recent evidence further suggests that colonization during key developmental windows may be linked to microbiota-dependent shifts in growth and immunity during undernutrition [22]. However, because our microbiota assessment was conducted at the genus level using qPCR, the observed association reflects *Clostridium* at the genus level only; species-specific composition, pathogenicity, enterotoxin production, and functional activity cannot be inferred from these data. Accordingly, mechanistic interpretations should be made cautiously, and the findings should be interpreted as an association rather than evidence of causation. Similar findings from Indonesian and other low-resource populations have linked microbial dysbiosis, including increased *Clostridium* and reduced phylogenetic diversity, with growth faltering and stunting [19,22,23]. These observations underscore the relevance of gut microbiota composition as a determinant of infant growth and metabolic health. Recent studies from Indonesia also report gut microbiota differences between children with impaired growth and those with normal growth, including findings from East Nusa Tenggara, Aceh, and urban low-income settings, supporting the relevance of microbiota-growth relationships in diverse Indonesian contexts [20,24,25]. Consistent with this, recent work also reports that stunting can be associated with persistent and potentially transferable gut microbiome alterations [26].

The predominance of beneficial bacteria in this population, alongside the observed negative effect of *Clostridium*, highlights the delicate balance between protective and pathogenic microbial communities in early life. Maintaining this balance is influenced by breastfeeding, maternal diet, hygiene, and environmental exposures. Previous trials have demonstrated that supplementation with human milk oligosaccharides promotes the growth of *Bifidobacterium* while suppressing opportunistic bacteria, resulting in improved gut maturation and growth outcomes [22,27]. Similarly, meta-analyses have shown that infant formulas supplemented with prebiotics or synbiotics beneficially modify gut microbial profiles and improve feeding tolerance and growth [28]. Furthermore, transitions from exclusive breastfeeding to mixed or complementary feeding have been shown to alter microbial diversity and metabolic activity, influencing growth trajectories during infancy [29]. The interaction between nutrition, immunity (eg, secretory IgA), and microbiota composition supports the need for integrated interventions that sustain gut health and optimal growth [30].

From a public health perspective, these findings emphasize that gut microbiota composition is not merely a biological variable but a potentially modifiable correlate of child growth and nutrition [17,22]. The observed association between *Clostridium* abundance and lower WAZ suggests that environmental and behavioral factors influencing microbial colonization, such as hygiene, breastfeeding practices, and maternal nutrition, represent actionable targets for community-based interventions [12,16,31]. The predominance of *Bifidobacterium* and *Lactobacillus* in well-nourished infants further reinforces the importance of promoting exclusive breastfeeding and dietary diversity during lactation [32-34]. Incorporating probiotic and prebiotic strategies into maternal and child nutrition programs could enhance resilience against microbial dysbiosis and strengthen early-life growth outcomes [35,36].

### Limitations

This study has several limitations. The cross-sectional design precludes causal inference, and temporality between gut microbiota composition and WAZ cannot be established. The modest sample size (n=88) may have limited statistical power to detect smaller associations, particularly in adjusted models; thus, nonsignificant findings should be interpreted cautiously. Because this analysis was nested within a prior randomized maternal supplementation trial, prenatal exposures—including supplementation adherence, maternal diet, and health during pregnancy—may have influenced infant gut microbiota through pathways not fully captured in the current models. Although supplementation allocation (MRJ vs MMS) was included as a covariate, residual confounding cannot be excluded. Maternal anthropometric data, including BMI at enrollment, were not available for this analysis and could not be adjusted for. Exclusive breastfeeding was included as a covariate; however, no dedicated subgroup analysis by breastfeeding status was conducted, and interpretations related to breastfeeding practices should therefore be made cautiously.

Furthermore, the use of qPCR quantification provides genus-level data and does not capture species-specific or functional microbial diversity. Future longitudinal studies using metagenomic and metabolomic approaches are warranted to elucidate the mechanistic pathways linking gut microbiota and early growth. Despite these limitations, this study provides locally relevant evidence from an understudied coastal population and highlights the potential role of *Clostridium* overabundance as a biomarker of growth faltering in early infancy.

### Conclusions and Recommendations

In this study of 6-month-old infants from coastal areas of Central Sulawesi, Indonesia, *Clostridium* was the only bacterial genus independently and inversely associated with WAZ, even after controlling for maternal, perinatal, and environmental factors. In contrast, *Bifidobacterium*, *Lactobacillus*, *Bacteroides*, and *E coli* showed no significant associations with WAZ.

These findings indicate that higher *Clostridium* abundance is associated with lower WAZ and highlight the importance of maintaining a balanced gut microbiota for optimal infant nutrition. Future longitudinal studies should further examine how breastfeeding practices, maternal nutrition during pregnancy and lactation, and microbiota-supportive approaches may influence infant gut microbiota composition and growth outcomes in coastal and resource-limited settings.

## Supplementary material

10.2196/87452 Multimedia Appendix 1 DNA extraction and quantitative polymerase chain reaction quantification of gut microbiota.

10.2196/87452 Checklist 1 STROBE statement.

## Abbreviations

CFU: colony-forming unit
MMS: multiple micronutrient supplements
MRJ: *Moringa oleifera* enriched with royal jelly
qPCR: quantitative polymerase chain reaction
STROBE: Strengthening the Reporting of Observational Studies in Epidemiology
WAZ: weight-for-age *z* score
WHO: World Health Organization
