# Supplementary material for: Clostridium Abundance and Lower Weight-for-Age z Scores Among 6-Month-Old Infants: Nested Cross-Sectional Study
Source: JMIR Pediatr Parent. 2026 Jul 6;9:e87452. doi: 10.2196/87452 (PMC13335944; doi:10.2196/87452)
Supplement: Multimedia Appendix 1 [file pediatrics-v9-e87452-s001.docx]

**Multimedia Appendix 2**

**DNA Extraction and qPCR Quantification of Gut Microbiota**

**Overview.**

This appendix provides additional methodological detail on stool DNA extraction and genus-level quantitative PCR (qPCR) used to quantify selected gut microbiota genera in six-month-old infants.

**Stool sample handling and storage**

Infant stool samples were collected during household visits and stored frozen (−20 °C) prior to laboratory processing. Samples were thawed on ice immediately before DNA extraction.

**DNA extraction (Presto™ Stool DNA Extraction Kit, Geneaid)**

Total DNA was extracted from stool using the Presto™ Stool DNA Extraction Kit (Geneaid Biotech Ltd., Taiwan) with bead beating and inhibitor-removal steps to minimize PCR inhibitors. Key steps and parameters are summarized below.

**1. Sample lysis and homogenization**

Transfer approximately 180–220 mg stool into a bead-beating tube containing ceramic beads. Add 800 µL lysis buffer (ST1), vortex briefly, then incubate at 70 °C for 5 minutes. Homogenize by horizontal vortexing at maximum speed for 10 minutes at room temperature. Centrifuge at ~8,000 ×g for 2 minutes to reduce foam and clarify lysate, then transfer 500 µL supernatant to a new tube.

**2. PCR inhibitor removal**

Add 150 µL inhibitor-removal buffer (ST2), vortex (~5 seconds), and incubate at 0–4 °C for 5 minutes. Centrifuge at ~16,000 ×g for 3 minutes. Load 500 µL clear supernatant onto an inhibitor-removal column and centrifuge at ~16,000 ×g for 1 minute. Retain the flow-through.

**3. DNA binding**

Add 800 µL binding buffer (ST3) to the flow-through and mix immediately. Load 700 µL onto a GD spin column and centrifuge at ~16,000 ×g for 1 minute; discard flow-through. Repeat with the remaining mixture until all is loaded.

**4. Wash and drying**

Wash the GD column with 400 µL ST3 buffer (centrifuge ~16,000 ×g for 30 seconds), then wash twice with 600 µL wash buffer (ethanol-added), centrifuging ~16,000 ×g for 30 seconds each. Dry the membrane by centrifuging at ~16,000 ×g for 3 minutes.

**5. Elution**

Transfer the dry column to a clean microcentrifuge tube. Add 30–100 µL elution buffer (preheated to 60 °C) to the center of the membrane, incubate ≥2 minutes, then centrifuge at ~16,000 ×g for 2 minutes to elute DNA. DNA was stored at −20 °C until qPCR.

**Genus-level qPCR quantification**

Genus-level qPCR was used to quantify five bacterial genera: *Bifidobacterium, Lactobacillus, Bacteroides, Clostridium,* and *Escherichia coli*. Assays used genus-specific primer sets targeting 16S rRNA gene regions. Results were reported as log10 CFU/mL equivalents based on standard curves generated from serial dilutions of appropriate standards.

Quality control procedures should include no-template controls, duplicate reactions (recommended), and melt-curve inspection (for SYBR-based assays) to verify specificity. If Ct values fall outside the linear range of the standard curve, samples should be re-assayed using an appropriate dilution.
