# Supplementary material for: Clostridium Abundance and Lower Weight-for-Age z Scores Among 6-Month-Old Infants: Nested Cross-Sectional Study
Source: JMIR Pediatr Parent. 2026 Jul 6;9:e87452. doi: 10.2196/87452 (PMC13335944; doi:10.2196/87452)
Supplement: Checklist 1 [file pediatrics-v9-e87452-s002.docx]

**Multimedia Appendix 1**

**STROBE Statement—Checklist of items that should be included in reports of cross-sectional studies**

| Section/Item | Item No | Recommendation | Manuscript location (page/section) |
| --- | --- | --- | --- |
| Title and abstract | 1(a) | Indicate the study’s design with a commonly used term in the title or the abstract | Title (p1); Abstract: Methods (p1) |
| Title and abstract | 1(b) | Provide in the abstract an informative and balanced summary of what was done and what was found | Abstract (p1–2) |
| Background/rationale | 2 | Explain the scientific background and rationale for the investigation being reported | Introduction (p2–4) |
| Objectives | 3 | State specific objectives, including any prespecified hypotheses | Introduction-Objectives/Aim (p3–4) |
| Study design | 4 | Present key elements of study design early in the paper | Methods: Study Design and Setting (p4) |
| Setting | 5 | Describe the setting, locations, and relevant dates | Methods: Study Design and Setting (p4) |
| Participants | 6(a) | Give eligibility criteria and methods of selection | Methods: Participants (p5); Figure 1 (p5–6) |
| Variables | 7 | Clearly define outcomes, exposures, confounders | Methods: Variables and Outcomes (p6) |
| Data sources/measurement | 8 | Describe data sources and measurement methods | Methods: Data Collection; Anthropometry; Microbiota (p6–8) |
| Bias | 9 | Describe efforts to address bias | Methods: Statistical Analysis (bias statement added); Discussion: Limitations (p18–19) |
| Study size | 10 | Explain how study size was determined | Methods: Sample size considerations (p6) |
| Quantitative variables | 11 | Explain handling of quantitative variables | Methods: Variables and Outcomes; Statistical Analysis (p6, p8–9) |
| Statistical methods | 12(a) | Describe all statistical methods | Methods: Statistical Analysis (p8–9) |
| Statistical methods | 12(b) | Describe subgroup/interaction analyses | Not applicable (no subgroup/interaction analyses) |
| Statistical methods | 12(c) | Explain how missing data were addressed | Methods: Statistical Analysis (no missing data) (p9) |
| Statistical methods | 12(d) | Describe sampling strategy if applicable | Not applicable (no complex sampling design) |
| Statistical methods | 12(e) | Describe sensitivity analyses | No formal sensitivity analyses; supplementation group included as covariate in all adjusted models (Methods: Statistical Analysis, p9) |
| Participants | 13(a) | Report numbers at each stage | Figure 1; Results: Participants (p9) |
| Participants | 13(b) | Give reasons for non-participation | Methods: Participants; Figure 1 (p5–6) |
| Participants | 13(c) | Use flow diagram | Figure 1 (p5–6) |
| Descriptive data | 14(a) | Give characteristics of participants | Results: Table 1 (p9–10) |
| Descriptive data | 14(b) | Indicate missing data | Methods: Statistical Analysis (no missing data) (p9) |
| Outcome data | 15 | Report outcome measures | Results: Table 1 (WAZ, underweight) (p9–10) |
| Main results | 16(a) | Give unadjusted and adjusted estimates | Results: Table 3; Figure 3 (p13–15) |
| Main results | 16(b) | Report category boundaries | Methods: Variables (WAZ < −2 SD) (p6) |
| Main results | 16(c) | Translate risk if relevant | Not applicable (continuous outcome analysis) |
| Other analyses | 17 | Report other analyses | ResultsTable 2; Figures 2–3; Wilcoxon signed-rank test (p12–14) |
| Key results | 18 | Summarise key findings | Discussion: Opening summary (p16) |
| Limitations | 19 | Discuss limitations and bias | Discussion: Limitations (p18–19) |
| Interpretation | 20 | Provide cautious interpretation | Discussion; Conclusions (p16–20) |
| Generalisability | 21 | Discuss external validity | Discussion: Public health implications (p18–20) |
| Funding | 22 | Provide funding information | Funding/Conflict of interest (p20) |
